# Hand Hygiene Knowledge and Self-Reported Hand Washing Behaviors among Restaurant Kitchen Chefs in Jiangsu Province, China

**DOI:** 10.3390/ijerph18042149

**Published:** 2021-02-22

**Authors:** Bin Cui, Shao Ying Li, Linda Dong-Ling Wang, Xiang Chen, Jun Ke, Yi Tian

**Affiliations:** 1 Business School of Yangzhou University, Yangzhou 225001, China; lsyyzdxzm@126.com (S.Y.L.); jke@yzu.edu.cn (J.K.); ytian@yzu.edu.cn (Y.T.); 2 Jiangsu Key Laboratory of Zoonosis, Yangzhou University, Yangzhou 225001, China; chenxiang@yzu.edu.cn; 3 Jiangsu Co-Innovation Center for Prevention and Control of Important Animal Infectious Diseases and Zoonoses, Yangzhou University, Yangzhou 225001, China; 4 Institute of Translational Medicine, Medical College, Yangzhou University, Yangzhou 225001, China; lindawdl@yzu.edu.cn; 5 Jiangsu Key Laboratory of Experimental & Translational Non-Coding RNA Research, Yangzhou University, Yangzhou 225001, China

**Keywords:** hand hygiene knowledge, hand washing, self-reported behaviors, restaurant kitchens, Chinese chefs

## Abstract

Inadequate hand washing among chefs is a major contributor to outbreaks of foodborne illnesses originating in restaurants. Although many studies have evaluated hand hygiene knowledge (HHK) and self-reported hand washing behaviors (HWBs) in restaurant workers in different countries, little is known about HHK and HWBs in restaurant kitchen chefs, particularly in China. In this study, we interviewed 453 restaurant kitchen chefs in Jiangsu Province in China regarding their HHK and HWBs and used Chi-square tests (Fisher exact tests), pairwise comparisons, and linear regression models to analyze the responses and identify determinants of HHK and HWBs. Results reveal that less frequent hand washing after leaving work temporarily and after touching used cutlery were the main issues among restaurant kitchen chefs in Jiangsu Province. Kitchen hands had lower levels of HHK and engaged less frequently in good HWBs than the other type of chefs. Furthermore, working in a large restaurant and having worked in the restaurant industry for a longer amount of time were correlated with better HHK and HWBs. These findings suggest that close attention should be paid to the HWBs of chefs during food preparation, that kitchen hands are the key group of restaurant kitchen workers who need training in HHK, and that regulatory activities should focus on small-scale restaurants.

## 1. Introduction

Foodborne diseases represent a substantial health burden and result in considerable morbidity and mortality globally [1]. In China, 2795 foodborne disease outbreaks were reported between 2003 and 2008, resulting in 62,559 illnesses, 31,261 hospitalizations, and 330 deaths [2]. More recently, China has faced various and unprecedented foodborne disease outbreaks originating from all points along the food chain [3].

Although contamination of food can occur at any point from farm to table, food workers are primarily responsible for foodborne disease outbreaks in many settings [4], and restaurant food workers are known to be a common source of foodborne illness [5]. Indeed, restaurants account for the highest percentage (around 40%) of foodborne disease outbreaks involving food service facilities in most developed countries [6]. Over half of all foodborne disease outbreaks reported to the Centers for Disease Control and Prevention in the United States are associated with eating at restaurants or delicatessens [7]. Furthermore, one study reported that 23.4% of foodborne disease incidents in China occur in commercial restaurants, which is second only to the percentage of cases that happen at home (24.4%) [8]. Therefore, understanding hand hygiene among restaurant kitchen chefs is of great significance for reducing the incidence of foodborne illness.

Among the causes of foodborne illness in the food service industry, inadequate hand washing has been found to be a major contributor [9,10]. Pathogens can easily be transferred from food workers’ bodies, as well as utensils and kitchen surfaces, to raw food during preparation [11,12]. Engaging in proper hand hygiene, either by cleaning one’s hands with soap and water or by using hand sanitizer, is the single most effective way for food workers to reduce the spread of preventable infectious diseases [13,14]. However, proper adherence to hygiene standards can vary greatly among restaurant workers and in different food preparation contexts.

Many studies have focused on food safety and hygiene issues in restaurants in countries other than China. In general, however, these studies have primarily addressed food safety knowledge and self-reported Hazard Analysis and Critical Control Points (HACCP) practices [15], food safety knowledge and practices [16,17,18,19,20,21,22,23,24], food allergy knowledge [25], and food hygiene and sanitation knowledge and practices [26,27]. Although some of these studies have investigated, in part, restaurant workers’ hand hygiene knowledge (HHK) and self-reported hand washing behaviors (HWBs), and one study has even focused on self-reported HWBs among food handlers certified under the FOODSAFE training program [28]. In China, recent research has primarily focused on safe food handling in households [29] and food safety knowledge and practices among food handlers in the coastal resort of Guangdong [30]. To our knowledge, few studies have specifically analyzed HHK and HWBs among restaurant kitchen chefs, particularly in China.

Chinese food is famous worldwide. Jiangsu cuisine, which is typical of Jiangsu Province in central China, is one of the eight famous regional Chinese cuisines [31]. Jiangsu cuisine is characterized by being freshly prepared by hand immediately prior to cooking. These traditional preparation conditions increase the risk of spreading foodborne disease due to poor HWBs among kitchen chefs.

Therefore, the objective of this study was to evaluate and identify determinants of HHK and HWBs among four types of chefs (pastry chefs, who are usually responsible for preparing wheat-based foods; cuisine chefs, who are usually responsible for steaming, frying, braising, boiling, and roasting; joint cuisine and pastry chefs who can carry out both types of tasks as needed; and kitchen hands, who are usually responsible for rough processing of fresh ingredients) in restaurant kitchens in Jiangsu Province, China. This cross-sectional study was conducted in the summer (July–August) of 2020, when increased numbers of cases of foodborne diseases are reported due to high temperatures. The results from this study will help food hygiene regulators develop customized educational programs and inspection routines to improve the level of hand hygiene among restaurants chefs.

## 2. Methods

### 2.1. Sampling

The current study was conducted in restaurants in seven of the 13 prefectural-level cities in Jiangsu Province, China. Two large restaurants (operating area greater than 500 m^2^), three medium-sized restaurants (operating area of 150–500 m^2^), and five small restaurants (operating area less than 150 m^2^) were randomly selected from each of the seven selected cities. In each selected restaurant, all kitchen chefs who were present at the time of the study were invited to participate. Five trained research assistants conducted face-to-face interviews with the chefs using a standardized questionnaire. A total of 453 subjects completed interviews.

### 2.2. Ethics, Consent, and Permissions

Ethical approval for the project was received from Yangzhou University prior to administering the survey.

To avoid disrupting the chefs’ work schedules and to improve the accuracy of the survey answers, the interviews were carried out during normal break times, such as between lunch and when the chefs started working again in the afternoon. The purpose of the study was explained, and then, the chefs’ consent to participate was sought. Those who agreed to participate completed a face-to-face interview using a standardized questionnaire. The questionnaire was fully anonymous and did not collect any personal identifying information.

### 2.3. Study Instrument

The questionnaire was divided into three sections. The first section was designed to collect socio-economic information including gender, age, level of education, income per month, marital status, type of work, years working at the current restaurant, years working in the restaurant industry, and scale of the restaurant.

The second part of the survey sought to establish a knowledge score (KS) for HHK and self-reported HWBs and included eight items adapted from the “Food safety operation standard for catering service” enacted by 2018 Department of Food Safety Supervision and Administration of the State Administration for Market Regulation of China. To understand the level of awareness regarding each knowledge item, we asked dichotomous (yes/no) questions using the following format: “Have you ever heard of…?”. To assess HWBs, we asked participants questions according to the following format: “How many times out of 10 would you say you wash your hands with soap and water or by using hand sanitizer?”, with responses ranging in frequency from 0 to 10 times. All interviews were conducted in Chinese. The items presented in English in this paper were translated and back-translated twice to ensure equivalent meanings.

The third part of the survey sought to measure the major constructs of the Theory of Planned Behavior (TPB), using items adapted from earlier pre-validated studies [32,33]. The TPB constructs included attitudes (five items), subjective norms (four items), and perceived behavioral controls (four items). The TPB constructs were measured by asking the respondents to indicate their agreement with a set of statements on a 7-point Likert-type scale (ranging from “1 = very strongly disagree” to “7 = very strongly agree”) (Table 1).

### 2.4. Data Analysis

The data were analyzed in three stages. First, Cronbach’s alpha (α) coefficients for each latent variable were calculated to assess the reliability of measures for TPB constructs using the IBM SPSS Statistics 20.0 software package (IBM company, Armonk, NY, USA), and Confirmatory Factor Analysis (CFA) was then conducted to assess the validity of measures for TPB constructs using Mplus 7.0 (Muthén & Muthén, Los Angeles, CA, USA).

Second, Chi-square tests (Fisher exact tests, if appropriate) and pairwise comparisons of multiple sample rates were used to compare the four groups of chefs in terms of HHK and self-reported HWBs (*p*-values of less than 0.05 were considered significant). The chefs’ HWBs were assigned a score of 0 and 10, with scores below 5 considered poor.

Third, linear regression was employed to examine the association between the socio-economic information, TPB constructs, KS, and self-reported HWBs. To facilitate subsequent linear regression analysis, the KSs were calculated based on the percentage of correct answers to eight questions. The three TPB and HWB dimensions were standardized by adding together the scores for all the items making up the factor (or dimension), subtracting the mean value, and dividing by the standard deviation. The level of significance was set at 95%.

## 3. Results

All but two of the restaurants indicated their willingness to participate in the study, for a response rate of 97.1% (68/70). To evaluate HHK and HWBs among restaurant chefs in Jiangsu Province, we administered a three-part survey to 453 workers from 68 restaurants. The demographic characteristics of the study participants are presented in Table 2. Of the respondents, most of the participants were male, under the age of 35, had less than a senior high school level of education, and earned less than 18,000 Chinese Yuan per month; 55.8% were married. Overall, 25.4% had been working in the current restaurant for less than 1 year, while 22.3% had worked for more than 6 years in the current restaurant. In addition, 13.7% of the respondents were pastry chefs, 52.1% were cuisine chefs, 11.3% were chefs of cuisine and pastry, and 23.0% were kitchen hands. Furthermore, 10.2% had worked in the restaurant industry for less than 1 year, and 26.5% had worked for more than 10 years in the restaurant industry. Finally, 30.9% of the chefs worked in a restaurant with an operating area of less than 150 m^2^, while 33.8% worked in a restaurant with an operating area of more than 500 m^2^. Taken together, these results suggest that we recruited a representative sample of kitchen chefs according to demographics.

Next, we assessed the reliability and validity of measures for the TPB constructs. After removing the item “It’s impossible for me to wash my hands before preparing food” from the perceived behavioral control constructs, all α values approached or exceeded 0.70 (see Table 1), indicating high internal consistency (internal reliability) for each measure [34]. The CFA results were as follows: χ^2^/df = 2.12, root mean square error of approximation (RMSEA) = 0.050, Tucker–Lewis Index (TLI) = 0.928, Comparative Fit Index (CFI) = 0.947, and standardized root mean square residual (SRMR) = 0.058. The values for CFI and TLI were greater than 0.9, χ^2^/df was less than 3.0, and RMSEA and SRMR were less than 0.08, suggesting an acceptable fit of the model to the data [35].

**Figure 1:**
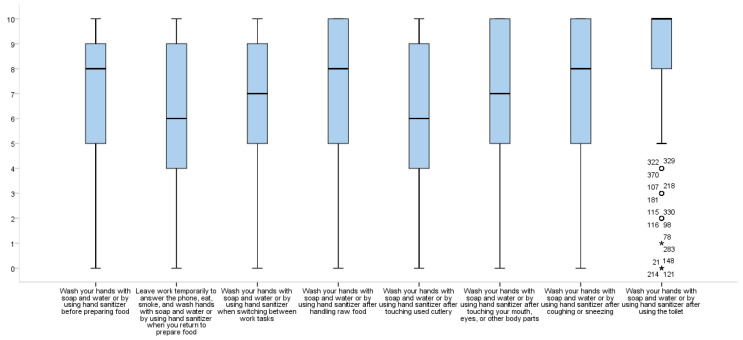
Self-reported hand washing behavior. (circle and asterisks represents samples with outliers).

Evaluation of HHK and HWBs among chefs showed that the respondents’ KS for “Hands should be washed with soap and water or by using hand sanitizer after touching used cutlery” (74.8%) were significantly lower than their KSs for other items, while KSs for “Hands should be washed with soap and water or by using hand sanitizer before preparing food” (97.4%) and “Hands should be washed with soap and water or by using hand sanitizer after using the toilet” (98.0%) were significantly higher than for other items (χ^2^ = 177.48, *p* < 0.001) (Table 3). Respondents generally reported lower adoption of “Leave work temporarily to answer the phone, eat, smoke, and wash hands with soap and water or by using hand sanitizer when you return to prepare food” (median = 6.00, possible range 1–10) and “Wash your hands with soap and water or by using hand sanitizer after touching used cutlery” (median = 6.00, possible range 1–10) compared with the other HWBs (Figure 1). The most commonly adopted HWB was “Wash your hands with soap and water or by using hand sanitizer after using the toilet” (87.4% of respondents reported engaging in this HWB more than 6–10 out of 10 times), followed by “Wash your hands with soap and water or by using hand sanitizer before preparing food” (74.4% did so 6–10 out of 10 times) (χ^2^ = 131.69, *p* < 0.001) (Table 4).

Regarding comparison of the four groups of chefs in terms of HHK and self-reported HWBs, the Fisher exact test results (Table 5) showed that the KSs for “Hands should be washed with soap and water or by using hand sanitizer before preparing food” (χ^2^ = 7.63, *p* < 0.05) and “Hands should be washed with soap and water or by using hand sanitizer after using the toilet” (χ^2^ = 5.67, *p* < 0.05) were significantly lower for kitchen hands than for the other kitchen chefs. The Chi-square tests and pairwise comparison results (Table 5) showed that kitchen hands’ KS for “Hands should be washed with soap and water or by using hand sanitizer after touching your mouth, eyes or other body parts” (χ^2^ = 8.23, *p* < 0.05) was significantly lower than that for cuisine and pastry chefs, and their KS for “Hands should be washed with soap and water or by using hand sanitizer after coughing or sneezing” (χ^2^ = 8.49, *p* < 0.05) was significantly lower than that for pastry chefs. The Chi-square tests and pairwise comparison results (Table 6) showed that the proportion of kitchen hands with the good HWBs “Leave work temporarily to answer the phone, eat, smoke, and wash hands with soap and water or by using hand sanitizer when you return to prepare food” (χ^2^ = 8.01, *p* < 0.05) and “Wash your hands with soap and water or by using hand sanitizer after touching your mouth, eyes or other body parts” (χ^2^ = 10.64, *p* < 0.05) was significantly lower than the proportion of cuisine and pastry chefs with good scores for these HWBs. In addition, the proportion of both kitchen hands and cuisine chefs with good scores for the HWB “Wash your hands with soap and water or by using hand sanitizer after coughing or sneezing” (χ^2^ = 12.74, *p* < 0.05) was significantly lower than that of pastry chefs.

Regarding the TPB constructs, respondents generally reported positive attitudes (mean value = 6.58 possible range 1–7) toward HWBs, while perceived behavioral control values (mean value = 5.41 possible range 1–7) were marginally lower (Table 1) among the three TPB constructs.

Regarding the determinants of HHK and HWBs, the results of the multivariate linear regression (Table 7) showed that the number of years of working in the restaurant industry and the scale of the restaurant were significantly and positively associated with KSs. KSs and attitudes were significantly and positively associated with self-reported HWBs, while restaurant scale was significantly and positively associated with both KSs and self-reported HWBs.

## 4. Discussion

The present study offers insights into HHK and self-reported HWB practices among different types of restaurant kitchen chefs in Jiangsu Province in China, as well as determinants of HHK and self-reported HWBs. The results showed that significant differences existed among different HHK and self-reported HWB items among restaurant kitchen chefs, as well as between different types of restaurant kitchen chefs. Small-scale restaurants could be the main points or origins of outbreaks of foodborne illnesses due to poor HWBs among chefs, and HHK training is an important measure that can be taken to change kitchen chef HWBs.

We found that, in general, restaurant kitchen chefs have higher KSs regarding “Hands should be washed with soap and water or by using hand sanitizer before preparing food” and “Hands should be washed with soap and water or by using hand sanitizer after using the toilet”. Similar findings have been reported for restaurant workers in Jordan [21], food workers in Ireland [36], food handlers in food service establishments in the United Arab Emirates [24], and food handlers in restaurants in Kuwait [17]. We also found that the KS for Chinese restaurant kitchen chefs regarding “Hands should be washed with soap and water or by using hand sanitizer after touching used cutlery” (74.8%) was significantly lower. This suggests that restaurant worker training regarding hand hygiene is broadly similar in different countries. 

Our results show that Chinese kitchen chefs wash their hands frequently after using the toilet, but that their hand washing frequency during food processing (e.g., after leaving work temporarily, after touching used cutlery) is lower. This is consistent with previous study conducted in Malaysia [37], suggesting that restaurants should strengthen hand washing supervision during the cooking process.

We further found that kitchen hands not only had significantly lower KSs regarding washing hands with water or hand sanitizer before preparing food, after touching their mouth, eyes, or other body parts, after coughing or sneezing, and after the using toilet, but also reported significantly lower rates of related HWBs. This observation may be related to the nature of their job. In general, kitchen hands in Chinese restaurants are responsible for the cleanliness of the kitchen and rough processing, such as livestock slaughter and vegetable cutting, cleaning, and sorting. Perhaps, because kitchen hands are not directly involved in the cooking process, their hand hygiene is easily neglected. However, the presence of pathogenic microorganisms on all food handlers’ hands makes them an important source of contamination that can be transferred from food to the mouth, nose, throat, and intestinal tract [38,39]. Indeed, even food handlers who are considered to be healthy host millions of pathogenic bacteria [39]. Thus, emphasis should be placed on training and hand washing supervision for kitchen hands in Chinese restaurants.

Chefs who had worked in the restaurant industry for many years were more likely to have higher hand hygiene KSs. This is inconsistent with a previous study from Jordan that found no association between food workers’ level of experience and total food safety KS [21], as well as a study conducted in Trinidad and Tobago that found that the length of employment in the foodservice industry had no significant impact on food safety knowledge [40]. The reason for this difference may be that the current study measured specific hand hygiene KSs rather than food safety.

The present study found no association between chefs’ socio-economic characteristics and hand hygiene KSs. This is consistent with a previous study of food handlers working in fast food restaurants in Jordan [21]. However, two studies from Malaysia reported different findings about education level and KS. For example, there was a significant difference in the mean personal hygiene KS among food handlers with different levels of education at primary schools in Hulu Langat district, Selangor [37], but no significant difference in food handlers’ food hygiene and sanitation knowledge depending on educational level among food handlers working in restaurants in Kuala Pilah, Malaysia [26]. This suggests that the association between the socio-economic characteristics of food handlers and their KSs may vary depending on the type of knowledge being measured.

This study found that the chefs from large-scale restaurants not only had higher hand hygiene KS but also had a higher frequency of self-reported HWBs. This is consistent with previous research that reported lower average knowledge and practice scores for food handlers employed in small businesses in Portugal [41]. This finding also reflects the greater emphasis that larger restaurants place on food hygiene training and supervision in China. In contrast, more attention should be paid to these issues in small-scale restaurants.

In our study, we found that chefs with a positive attitude to hand hygiene and higher KSs were more likely to report higher frequency of HWBs, which is consistent with previous studies of among food handlers working in restaurants in Malaysia [26], food handlers in Selangor [37], Finnish restaurant business operators [27], food handlers in restaurants in Saudi Arabia [18], and food handlers in restaurants in Kuwait [17]. A positive attitude is crucial in making the transition from safety knowledge to effective hygienic practices [42], because attitude is the “mediator between knowledge and practices” [42,43]. Years worked in industry were the significant contributors to knowledge score, and knowledge score was the strongest contributor to HWBs; however, years worked in industry was not a significant predictor of HWBs. Thus, it may be attitude that plays an important role, but the exact reasons need to be examined.

However, we found no association between subjective norm, perceived behavioral control, and self-reported HWBs. This is inconsistent with a previous study that identified subjective norms and perceived behavioral control as significant predictors of poor hand hygiene practices among caterers [44]. This difference may be related to the type of behaviors assessed, as Clayton and Griffith (2008) measured poor practices. Another possible reason for this apparent discrepancy is different management types. As Faour-Klingbeil, Kuri, and Todd (2015) found, management type is an integral element of TPB that influences food handlers’ practices [45]. Therefore, future research will need to consider management type.

This study had several limitations. First, the cross-sectional design excluded causal inference. However, the correlation among the different variables discussed in this study suggests that the data offer a plausible explanation for cause and effect in this population. Second, this research was limited to restaurant chefs in Jiangsu Province and therefore cannot be considered representative for chefs throughout China. However, the practice of freshly preparing Jiangsu cuisine immediately prior to consumption is also common in the other seven famous regional Chinese cuisines. Therefore, our results regarding HHK and self-reported HWBs is likely to be representative of other regions in China. Third, self-reported data are a cognitive measurement that is prone to egocentrism and cognitive bias [46] and often underestimates the magnitude of undesirable food handling practices [20]. Nonetheless, many studies have used self-reported methods because of the relatively low cost of implementation [17,22,37,42,46]. In the future, surveys based on observations of HWBs are needed.

## 5. Conclusions

In this study, we found that less frequent hand washing after leaving work temporarily and after touching used cutlery were the main issues among restaurant kitchen chefs in Jiangsu Province. Therefore, close attention should be paid to the HWBs of chefs during food preparation. Kitchen hands are the key group in Chinese restaurant kitchen workers who require training in hand hygiene as well as inspection regarding their HWBs.

Our findings indicate that small-scale restaurants could be the main risk sources of foodborne illnesses due to the poor HWBs exhibited by chefs in these kitchens. Therefore, regulators should focus on small-scale restaurants in China.

KSs and attitudes were both associated with kitchen chef HWBs in our study, suggesting that HHK training is an important factor that can change the HWBs of kitchen chefs. Changing chefs’ attitudes towards hand hygiene is another key element that could be leveraged to improve the level of restaurant hygiene. Taken together, in addition to strengthening education to transform chefs’ attitudes toward hand hygiene, implementing appropriate incentives and penalties to help chefs develop a positive attitude toward hand hygiene may be another feasible method to motivate chefs to engage in appropriate hand hygiene practices in restaurant kitchens.

## Figures and Tables

**Table 1:** Analysis of different dimensions of Protection Motivation Theory.

Variables	Items	M	SD	Cronbach’s α
Attitude				
V1	I consider it a good habit to wash my hands before getting ready to prepare food.	6.62	0.884	0.784
V2	I consider it sanitary to wash my hands before getting ready to prepare food.	6.57	0.945	
V3	Washing hands before getting ready to prepare food is an effective method for preventing the spread of disease.	6.53	0.947	
V4	I consider it a responsible gesture toward guests if we wash our hands before getting ready to prepare food.	6.65	0.926	
V5	I consider it useful to wash my hands before getting ready to prepare food.	6.53	0.958	
Subjective norms				
V1	Most people whose opinions I value think I should wash my hands whenever I am at work.	5.95	1.576	0.823
V2	Most people who are important to me think I should wash my hands whenever I am at work.	5.81	1.560	
V3	Most people whose opinion I value would wash their hands whenever they while at work if they had my job.	5.81	1.554	
V4	Most people who are important to me would wash their hands whenever they should while at work if they had my job.	5.88	1.471	
Perceived behavioral control				
V1	It is mostly up to me to wash my hands whenever I should while at work.	5.53	1.978	0.780
V2	If I want to, I can wash my hands whenever I should while at work.	5.32	2.097	
V3	I have complete control over whether I wash my hands whenever I should while at work.	5.39	1.878	

M: mean, SD: standard deviation.

**Table 2:** Respondent characteristics (N = 453).

Characteristics	N	%
Gender		
Female	87	19.2
Male	366	80.8
Age		
≤20 years	58	12.8
21–25 years	140	30.9
26–35 years	165	36.4
36–45 years	77	17.0
≥46 years	13	2.9
Education		
Primary or below	12	2.6
Junior high school	174	38.4
Senior high school	187	41.3
Three-year college	45	9.9
Undergraduate college or above	35	7.7
Years working at the current restaurant		
≤1 year	115	25.4
2 years	98	21.6
3 years	65	14.3
4 years	36	7.9
5 years	38	8.4
≥6 years	101	22.3
Years working in the restaurant industry		
≤1 year	46	10.2
2–3 years	95	21.0
4–5 years	98	21.6
6–7 years	49	10.8
8–9 years	45	9.9
≥10 years	120	26.5
Income per month (Chinese Yuan)		
≤5000	5	1.1
5001–8000	85	18.8
8001–12,000	149	32.9
12,001–15,000	89	19.6
15,001–18,000	65	14.3
18,001–21,000	28	6.2
≥21,001	32	7.1
Marital Status		
Unmarried	200	44.2
Married	253	55.8
Type of work in kitchen		
Pastry chef	62	13.7
Cuisine chef	236	52.1
Chef of cuisine and pastry	51	11.3
Kitchen hand	104	23.0
Restaurant scale (Operating area)		
≤150 m^2^	140	30.9
150–500 m^2^	160	35.5
≥500 m^2^	153	33.8

**Table 3:** Chi-square analysis of scores for different hand hygiene knowledge items.

Items	Answers *n* (%)	
	No	Yes
1. Have you ever heard that hands should be washed with soap and water or by using hand sanitizer before preparing food?	12 _a_ (2.6)	441 _a_ (97.4)
2. Have you ever heard that, after leaving work temporarily to answer the phone, eat, or smoke, hands should be washed with soap and water or by using hand sanitizer before returning to prepare food?	58 _b,c_ (12.8)	395 _b,c_ (87.2)
3. Have you ever heard that hands should be washed with soap and water or by using hand sanitizer when switching between work tasks?	77 _c,d_ (17.0)	376 _c,d_ (83.0)
4. Have you ever heard that hands should be washed with soap and water or by using hand sanitizer after handling raw food?	54 _b,c_ (11.9)	399 _b,c_ (88.1)
5. Have you ever heard that hands should be washed with soap and water or by using hand sanitizer after touching used cutlery?	114 _d_ (25.2)	339 _d_ (74.8)
6. Have you ever heard that hands should be washed with soap and water or by using hand sanitizer after touching your mouth, eyes, or other body parts?	81 _c,d_ (17.9)	372 _c,d_ (82.1)
7. Have you ever heard that hands should be washed with soap and water or by using hand sanitizer after coughing or sneezing?	44 _b_ (9.7)	409 _b_ (90.3)
8. Have you ever heard that hands should be washed with soap and water or by using hand sanitizer after using the toilet?	9 _a_ (2.0)	444 _a_ (98.0)
χ^2^	177.48	
*p* value	0.000	

_a,b,c,d_: Differences between values indicated by different subscript letters are statistically significant at the 0.05 level.

**Table 4:** Chi-square analysis of self-reported hand washing behaviors (HWBs).

Items	HWBs n (%)	
	0–5 Times	6–10 Times
1. Wash your hands with soap and water or by using hand sanitizer before preparing food.	116 _a_ (25.6)	337 _a_ (74.4)
2. Leave work temporarily to answer the phone, eat, smoke, and wash hands with soap and water or by using hand sanitizer when you return to prepare food.	183 _b,c_ (40.4)	270 _b,c_ (59.6)
3. Wash your hands with soap and water or by using hand sanitizer when switching between work tasks.	147 _a,b,c,d,e_ (32.5)	306 _a,b,c,d,e_ (67.5)
4. Wash your hands with soap and water or by using hand sanitizer after handling raw food.	139 _a,c,e_ (30.7)	314 _a,c,e_ (69.3)
5. Wash your hands with soap and water or by using hand sanitizer after touching used cutlery.	192 _b_ (42.4)	261 _b_ (57.6)
6. Wash your hands with soap and water or by using hand sanitizer after touching your mouth, eyes, or other body parts.	163 _b,c,d,e_ (36.0)	290 _b,c,d,e_ (64.0)
7. Wash your hands with soap and water or by using hand sanitizer after coughing or sneezing.	127 _a,b,e_ (28.0)	326 _a,d,e_ (72.0)
8. Wash your hands with soap and water or by using hand sanitizer after using the toilet.	57 _f_ (12.6)	396 _f_ (87.4)
χ^2^	131.69	
*p* value	0.000	

_a,b,c,d.e.f_: Differences between values indicated by different subscript letters are statistically significant at the 0.05 level.

**Table 5:** Chi-square analysis of knowledge scores regarding hand hygiene.

Items	Type of Work in Kitchen				χ^2^	*p* Value
	Pastry Chef n (%)	Cuisine Chef n (%)	Chef of Cuisine and Pastry n (%)	Kitchen Hand n (%)		
1. Hands should be washed with soap and water or by using hand sanitizer before preparing food						
No	0 (0)	4 (1.7)	0 (0)	8 (7.7)	7.63 *	0.010
Yes	62 (100)	232 (98.3)	51 (100)	96 (92.3)		
2. Leave work temporarily to answer the phone, eat, or smoke, and hands should be washed with soap and water or by using hand sanitizer when you return to prepare food						
No	8 _a_ (12.9)	25 _a_ (10.6)	8 _a_ (15.7)	17 _a_ (16.3)	2.58	0.461
Yes	54 _a_ (87.1)	211 _a_ (89.4)	43 _a_ (84.3)	87 _a_ (83.7)		
3. Hands should be washed with soap and water or by using hand sanitizer when alternated work						
No	8 _a_ (12.9)	37 _a_ (15.7)	6 _a_ (11.8)	26 _a_ (25.0)	6.77	0.081
Yes	54 _a_ (87.1)	199 _a_ (84.3)	45 _a_ (88.2)	78 _a_ (75.0)		
4. Hands should be washed with soap and water or by using hand sanitizer after handling raw food						
No	9 _a_ (14.5)	28 _a_ (11.9)	6 _a_ (11.8)	11 _a_ (10.6)	0.58	0.901
Yes	53 _a_ (85.5)	208 _a_ (88.1)	45 _a_ (88.2)	93 _a_ (89.4)		
5. Hands should be washed with soap and water or by using hand sanitizer after touching used cutlery						
No	16 _a_ (25.8)	53 _a_ (22.5)	13 _a_ (25.5)	32 _a_ (30.8)	2.67	0.445
Yes	46 _a_ (74.2)	183 _a_ (77.5)	38 _a_ (74.5)	72 _a_ (69.2)		
6. Hands should be washed with soap and water or by using hand sanitizer after touching your mouth, eyes, or other body parts						
No	13 _a,b_ (21.0)	40 _a,b_ (16.9)	3 _b_ (5.9)	25 _a_ (24.0)	8.23	0.042
Yes	49 _a,b_ (79.0)	196 _a,b_ (83.1)	48 _b_ (94.1)	79 _a_ (76.0)		
7. Hands should be washed with soap and water or by using hand sanitizer after coughing or sneezing						
No	1 _a_ (1.6)	22 _a,b_ (9.3)	5 _a,b_ (9.8)	16 _b_ (15.4)	8.49	0.037
Yes	61 _a_ (98.4)	214 _a,b_ (90.7)	46 _a,b_ (90.2)	88 _b_ (84.6)		
8. Hands should be washed with soap and water or by using hand sanitizer after using the toilet						
No	0 (0)	3 (1.3)	0 (0)	6 (5.8)	5.67 *	0.026
Yes	62 (100)	233 (98.7)	51 (100)	98 (94.2)		

_a,b_: Differences between values indicated by different subscript letters are statistically significant at the 0.05 level; * Fisher exact test.

**Table 6:** Chi-square analysis of self-reported hand washing behaviors.

Items	Type of Work in Kitchen				χ^2^	*p* Value
	Pastry Chef n (%)	Cuisine Chef n (%)	Chef of Cuisine and Pastry n (%)	Kitchen Hand n (%)		
1. Wash your hands with soap and water or by using hand sanitizer before preparing food						
0–5 times	11 _a_ (17.7)	63 _a_ (26.7)	11 _a_ (21.6)	31 _a_ (29.8)	3.56	0.313
6–10 times	51 _a_ (82.3)	173 _a_ (73.3)	40 a (78.4)	73 _a_ (70.2)		
2. Leave work temporarily to answer the phone, eat, or smoke, and wash hands with soap and water or by using hand sanitizer when you return to prepare food						
0–5 times	24 _a,b_ (38.7)	95 _a,b_ (40.3)	13 _b_ (25.5)	51 _a_ (49.0)	8.01	0.046
6–10 times	38 _a,b_ (61.3)	141 _a,b_ (59.7)	38 _b_ (74.5)	53 _a_ (51.0)		
3. Wash your hands with soap and water or by using hand sanitizer when alternating work						
0–5 times	17 _a_ (27.4)	84 _a_ (35.6)	11 _a_ (21.6)	35 _a_ (33.7)	4.60	0.203
6–10 times	45 _a_ (72.6)	152 _a_ (64.4)	40 _a_ (78.4)	69 _a_ (66.3)		
4. Wash your hands with soap and water or by using hand sanitizer after handling raw food						
0–5 times	22 _a_ (35.5)	70 _a_ (29.7)	10 _a_ (19.6)	37 _a_ (35.6)	4.90	0.179
6–10 times	40 _a_ (64.5)	166 _a_ (70.3)	41 _a_ (80.4)	67 _a_ (64.4)		
5. Wash your hands with soap and water or by using hand sanitizer after touching used cutlery						
0–5 times	25 _a_ (40.3)	100 _a_ (42.4)	17 _a_ (33.3)	50 _a_ (48.1)	3.20	0.362
6–10 times	37 _a_ (59.7)	136 _a_ (57.6)	34 _a_ (66.7)	54 _a_ (51.9)		
6. Wash your hands with soap and water or by using hand sanitizer after touching your mouth, eyes, or other body parts						
0–5 times	17 _a,b_ (27.4)	92 _a,b_ (39.0)	10 _b_ (19.6)	44 _a_ (42.3)	10.64	0.014
6–10 times	45 _a,b_ (72.6)	144 _a,b_ (61.0)	41 _b_ (80.4)	60 _a_ (57.7)		
7. Wash your hands with soap and water or by using hand sanitizer after coughing or sneezing						
0–5 times	9 _a_ (14.5)	75 _b_ (31.8)	8 _a,b_ (15.7)	35 _b_ (33.7)	12.74	0.005
6–10 times	53 _a_ (85.5)	161 _b_ (68.2)	43 _a,b_ (84.3)	69 _b_ (66.3)		
8. Wash your hands with soap and water or by using hand sanitizer after the using toilet						
0–5 times	7 _a_ (11.3)	31 _a_ (13.1)	6 _a_ (11.8)	13 _a_ (12.5)	0.19	0.979
6–10 times	55 _a_ (88.7)	205 _a_ (86.9)	45 _a_ (88.2)	91 _a_ (87.5)		

_a,b_: Differences between values indicated by different subscript letters are statistically significant at the 0.05 level.

**Table 7:** Multiple regression coefficients (standardized β, standard error) for socio-economic, knowledge score and Theory of Planned Behavior variables associated with self-reported hand washing behaviors.

Predictor Variables	Knowledge Score					Self-Reported Hand Washing Behavior				
	β	SE	95% Confidence Interval		VIF	β	SE	95% Confidence Interval		VIF
			Lower Bound	Upper Bound				Lower Bound	Upper Bound	
Demographic variable										
Gender (Male)	0.029	0.132	−0.186	0.334	1.256	0.024	0.123	−0.181	0.303	1.276
Age	−0.018	0.077	−0.169	0.133	2.711	0.115	0.071	−0.022	0.257	2.713
Level of education	0.049	0.054	−0.053	0.157	1.148	0.029	0.050	−0.067	0.129	1.162
Income	−0.067	0.044	−0.131	0.041	1.910	−0.013	0.041	−0.089	0.071	1.921
Marital status	−0.048	0.124	−0.340	0.149	1.768	0.081	0.115	−0.061	0.392	1.775
Years worked at the current restaurant	−0.040	0.033	−0.085	0.044	1.819	0.026	0.031	−0.046	0.074	1.827
Years worked in the restaurant industry	0.184 *	0.046	0.014	0.195	2.987	−0.123	0.043	−0.156	0.013	3.027
Restaurant size	0.109 *	0.059	0.019	0.249	1.025	0.116 *	0.058	0.032	0.261	1.184
Knowledge score	-	-	-	-	-	0.370 **	0.045	0.290	0.466	1.068
Major constructs of the Theory of Planned Behavior										
Attitude	no data	no data	no data	no data	no data	0.130 *	0.048	0.041	0.228	1.168
Subjective norm	no data	no data	no data	no data	no data	0.017	0.051	−0.084	0.119	1.463
Perceived behavioral control	no data	no data	no data	no data	no data	−0.004	0.051	−0.104	0.096	1.405

* *p* < 0.05, ** *p* < 0.01. “-” indicates no data. β, standardized coefficient; SE, standard error; VIF, variance inflation factor.

## Data Availability

The data presented in this study are available on reasonable request from the corresponding author. The data are not publicly available due to ethical requirements.
